# Supplementary material for: Maturation-dependent changes in the size, structure and seeding capacity of Aβ42 amyloid fibrils
Source: Commun Biol. 2024 Feb 6;7:153. doi: 10.1038/s42003-024-05858-7 (PMC10847148; doi:10.1038/s42003-024-05858-7)
Supplement: Supplementary file 1 — Supplementary Information [file 42003_2024_5858_MOESM1_ESM.pdf]

Supplementary Information for:

Maturation-dependent changes in the size, structure and seeding capacity of Aβ42 amyloid fibrils

Alyssa Miller[1], Sean Chia[1], Ewa Klimont[1], Tuomas P.J. Knowles[1,2]*, Michele Vendruscolo[1,]*, Francesco Simone Ruggeri[3,4]*

[1]*Yusuf Hamied Department of Chemistry, University of Cambridge, Lensfield Road, Cambridge, CB2 1EW, United Kingdom*

[2]*Cavendish Laboratory, University of Cambridge, Cambridge, CB3 0HE, United Kingdom*

[3]*Laboratory of Organic Chemistry, Stippeneng 4, 6703 WE, Wageningen University & Research, the Netherlands*

[4]*Physical Chemistry and Soft Matter, Stippeneng 4, 6703 WE, Wageningen University & Research, 6703 WE, Wageningen University, the Netherlands*

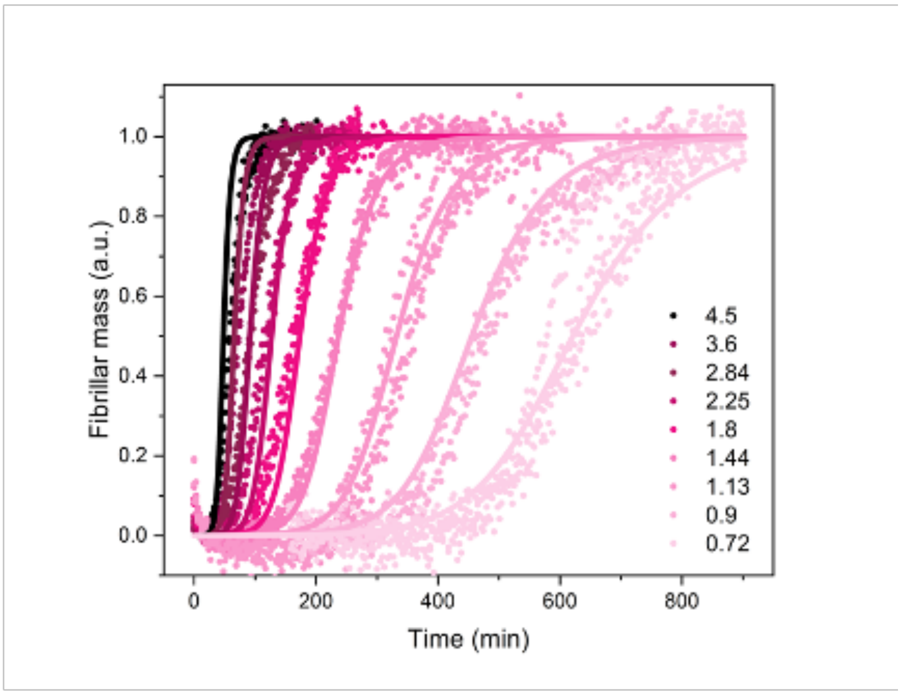

Figure S1. Aggregation assays were performed at a fixed seed concentration and with a varying monomer concentration. Aggregation curves were fit well to a secondary nucleation model (solid line), revealing that this is the dominant mechanism in the presence of seeds.

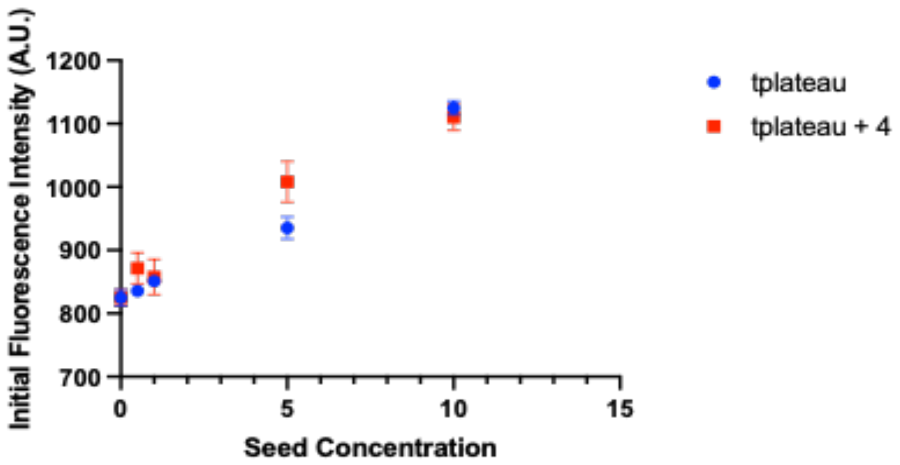

Figure S2. Initial aggregate mass of seeding stocks from early (0h) and late (4h) time points in the plateau was measured via ThT fluorescence intensity.

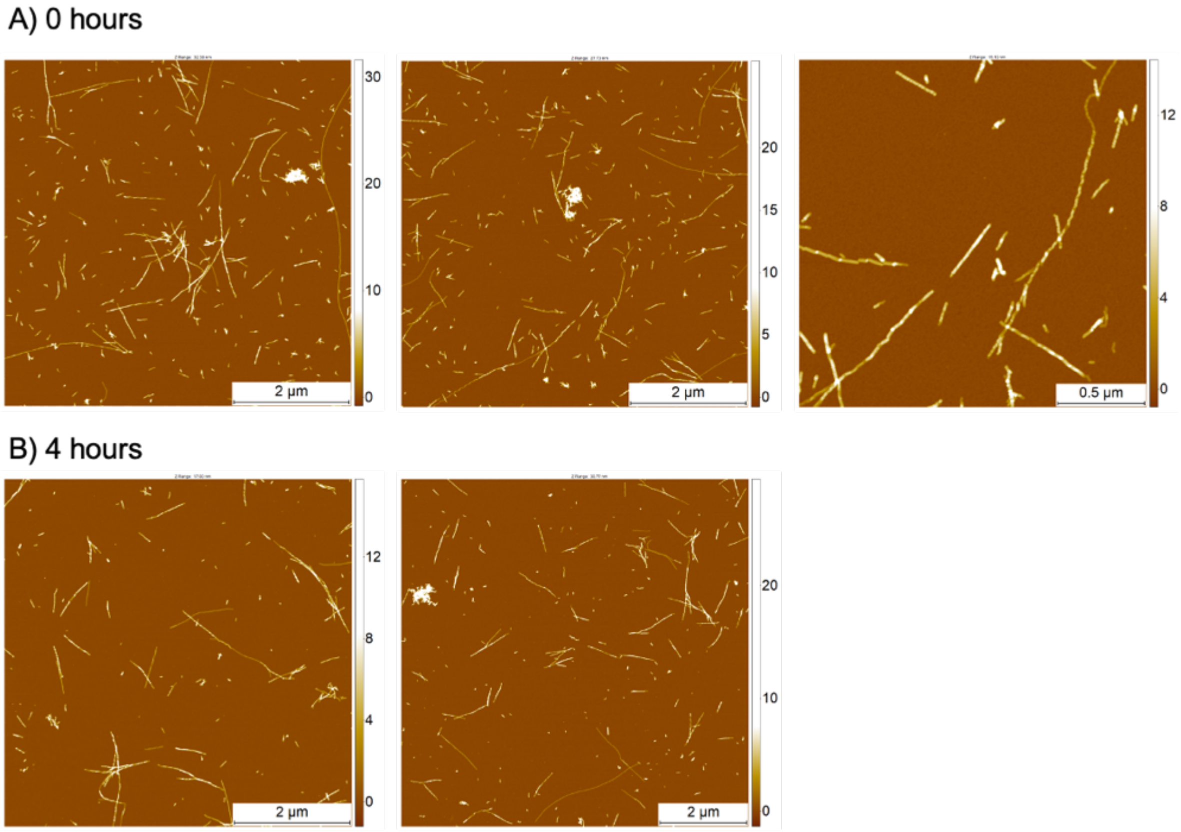

Figure S3. Example AFM images of samples acquired at a) 0 and b) 4 hour time points in the plateau phase.

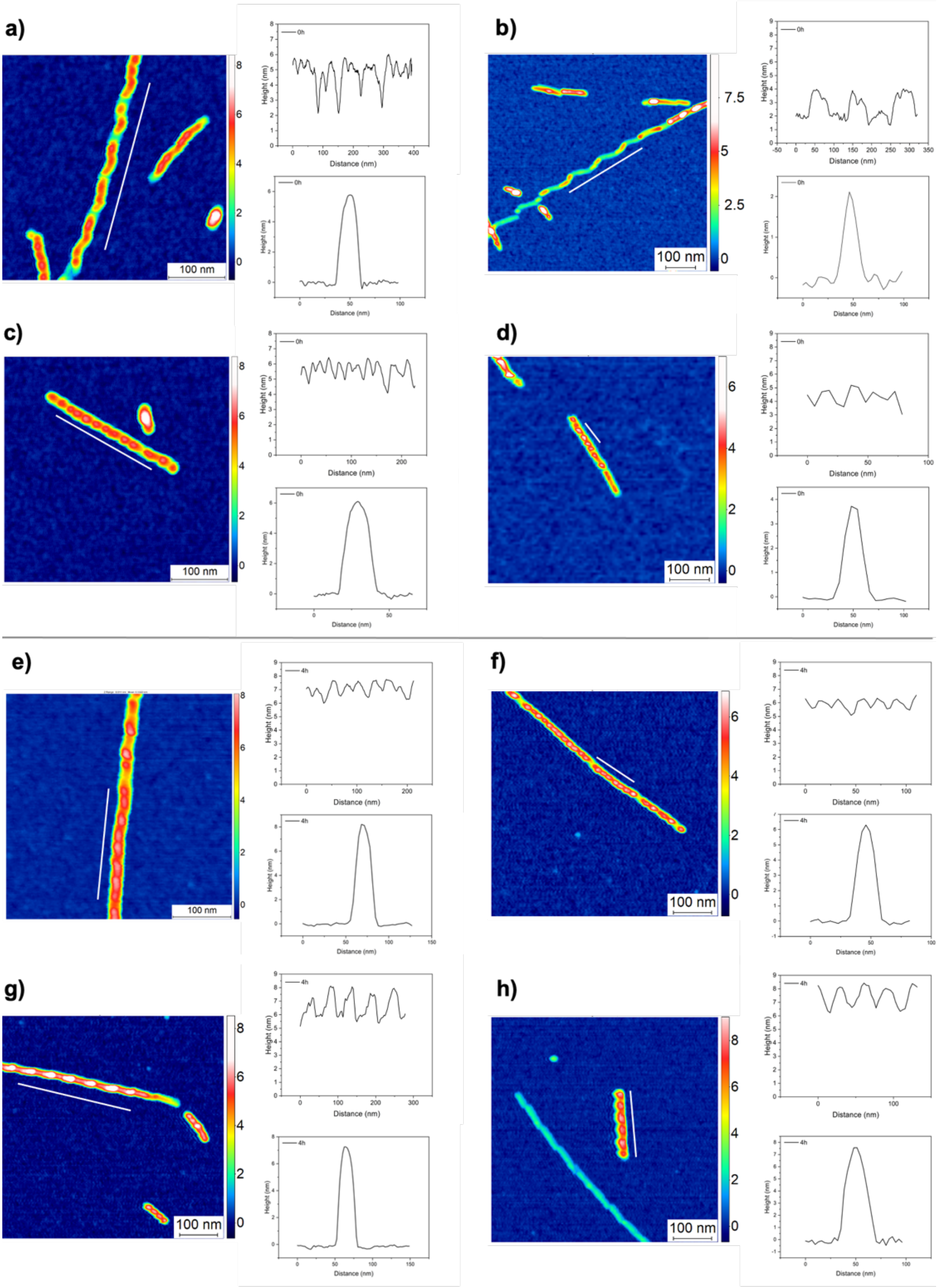

Figure S4. (a-h) Example AFM images of individual fibrils revealing well-defined twist morphology at 0h (a-d) and 4h (e-h) in the plateau phase. For each image, cross-sectional profiles are shown along the fibril axis to demonstrate the twist, and the fibril height, taken perpendicular to the fibril axis.

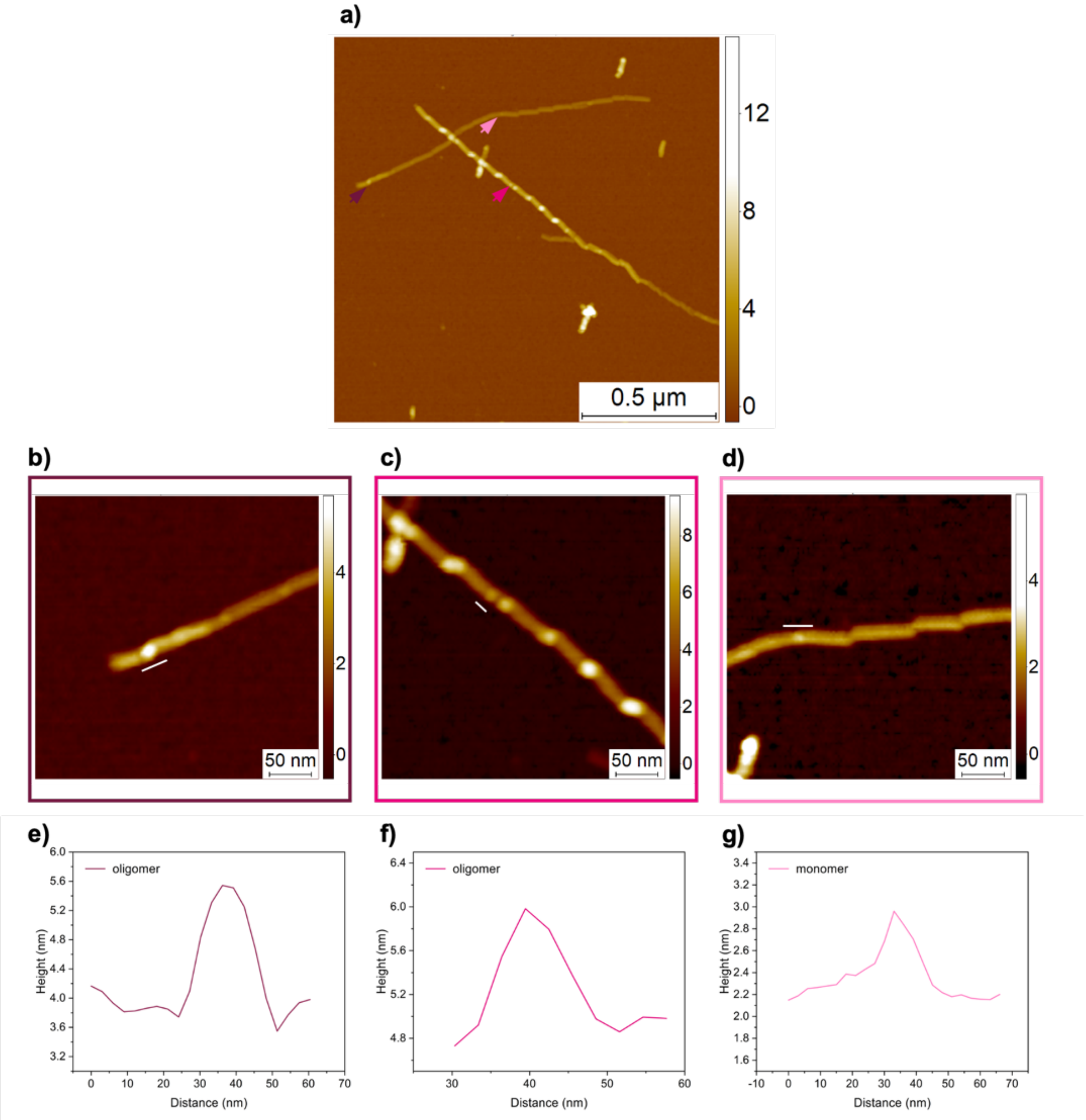

Figure S5. (a) High resolution AFM imaging reveals monomers and small oligomers adsorbed to fibril surfaces, indicated by coloured arrows. (b-d) Zooms of species adsorbed to fibril surfaces are shown, corresponding to arrows in panel a. (e-g) Height cross-sections were taken of individual species (location on image indicated by a white line).

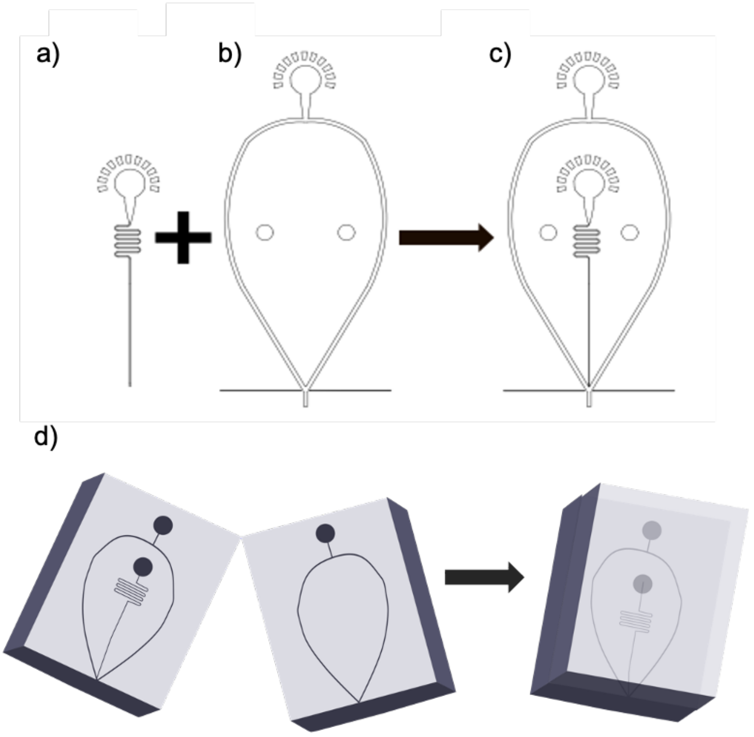

Figure S6. Schematic of the microfluidic device assembly. A 25 µm high liquid channel (a) and a 50 µm high gas channel (b) are combined in a two-step lithography process (c). Masters are used to fabricate two complementary PDMS slabs, which are carefully aligned and bonded to form the 3D spray nozzle device (d).

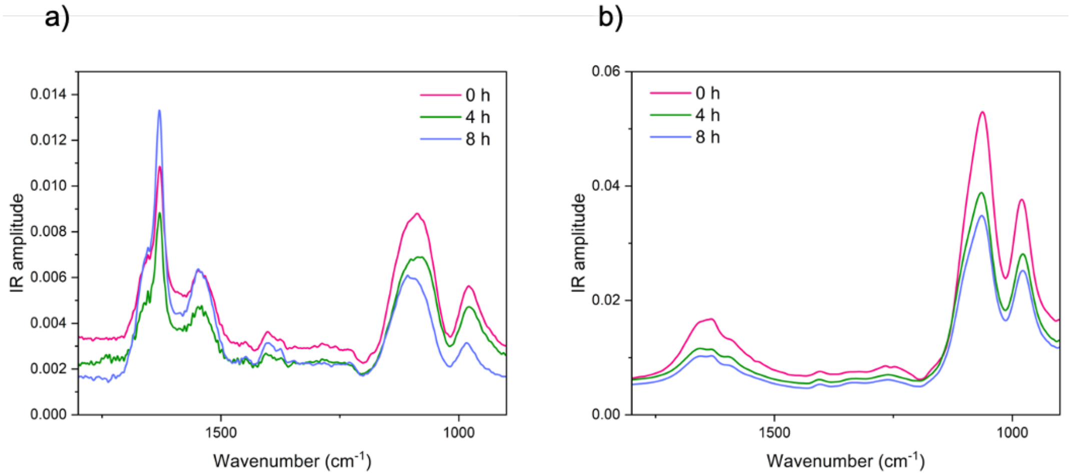

Figure S7. Non-normalised spectra of Aβ42 fibrils at 5mM in sodium phosphate buffer are shown for spray deposition (a) and manual deposition (b) to demonstrate the difference in spectroscopic sensitivity between sample preparation methods. Ultra-fast drying of picolitre-volume droplets from spraying results in reduced time for salt crystals to form. Salt crystallisation can be monitored by the peaks at ~1070 cm^{-1}. The intensity of the amide I peak is higher than that of the salt peak when deposited via spray. On the other hand, the large contribution of salt to the spectrum for manual deposition masks the protein amide I and II peaks, preventing secondary structure analysis.

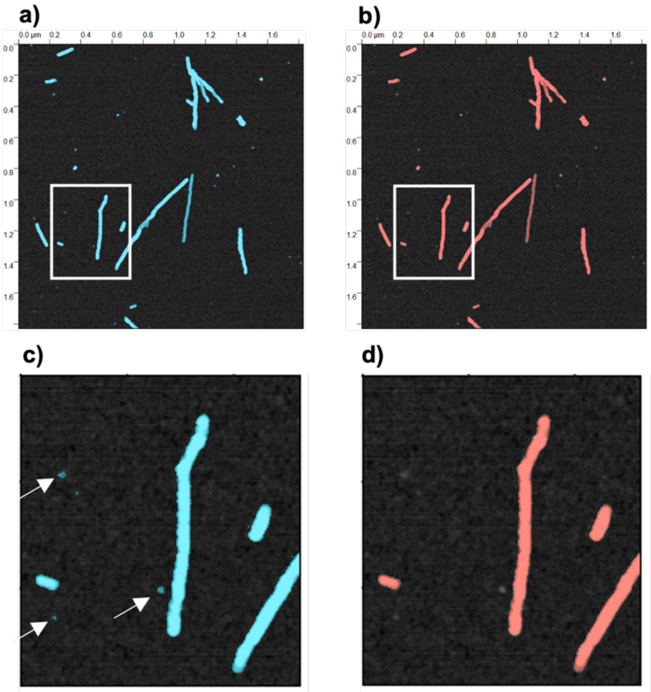

Figure S8. Relative volumes of fibrils vs monomers and small aggregates were measured from AFM maps. A height threshold was applied to consider all species, including monomers, oligomers and fibrils (blue) (a). Then, the height threshold was changed to only consider fibrillar species (red) (b). The volume of the selected species was then measured in both instances, and the volume of fibrils (red) was subtracted from the volume of all species, i.e. monomers, oligomers, and fibrils (blue) to determine the relative volumes of monomers and oligomers vs fibrils. Zooms are shown to further demonstrate the selection of monomers/oligomers vs fibrils, zoom area indicated by white boxes (c,d).
